# Deep Learning for Automatic Detection of Periodic Limb Movement Disorder Based on Electrocardiogram Signals

**DOI:** 10.3390/diagnostics12092149

**Published:** 2022-09-03

**Authors:** Erdenebayar Urtnasan, Jong-Uk Park, Jung-Hun Lee, Sang-Baek Koh, Kyoung-Joung Lee

**Affiliations:** 1 Artificial Intelligence Big Data Medical Center, Wonju College of Medicine, Yonsei University, Wonju 26426, Korea; 2 Department of Medical Artificial Intelligence, College of Medical Engineering, Konyang University, Daejeon 35365, Korea; 3 Department of Emergency Medicine, Wonju College of Medicine, Yonsei University, Wonju 26426, Korea; 4 Department of Preventive Medicine, Wonju College of Medicine, Yonsei University, Wonju 26426, Korea; 5 Department of Biomedical Engineering, College of Software and Digital Healthcare Convergence, Yonsei University, Wonju 26493, Korea

**Keywords:** deep learning, electrocardiogram, periodic limb movement syndrome, convolutional neural network, long short-term memory

## Abstract

In this study, a deep learning model (deepPLM) is shown to automatically detect periodic limb movement syndrome (PLMS) based on electrocardiogram (ECG) signals. The designed deepPLM model consists of four 1D convolutional layers, two long short-term memory units, and a fully connected layer. The Osteoporotic Fractures in Men sleep (MrOS) study dataset was used to construct the model, including training, validating, and testing the model. A single-lead ECG signal of the polysomnographic recording was used for each of the 52 subjects (26 controls and 26 patients) in the MrOS dataset. The ECG signal was normalized and segmented (10 s duration), and it was divided into a training set (66,560 episodes), a validation set (16,640 episodes), and a test set (20,800 episodes). The performance evaluation of the deepPLM model resulted in an F1-score of 92.0%, a precision score of 90.0%, and a recall score of 93.0% for the control set, and 92.0%, 93.0%, and 90.0%, respectively, for the patient set. The results demonstrate the possibility of automatic PLMS detection in patients by using the deepPLM model based on a single-lead ECG. This could be an alternative method for PLMS screening and a helpful tool for home healthcare services for the elderly population.

## 1. Introduction

Periodic limb movement syndrome (PLMS) is a repetitive, transient movement caused by specific muscle tension during sleep. PLMS episodes are repeated four or more times in a row, with each episode in the range of 0.5–5 s in duration. The interval between two episodes is 5–90 s. A frequent occurrence of PLMS (etc., a high number of average PLMSs per hour) can lead to arousal or sleep fragmentation that can affect health and well-being [1]. PLMS is also known to be associated with several other disorders, including cardiovascular disease, hypertension, depression, narcolepsy, rapid eye movement disorders, and Parkinson’s disease [2,3,4,5,6]. Therefore, early and simple diagnosis of PLMS is clinically important to prevent other related diseases.

Nocturnal polysomnography (PSG) is the standard diagnostic test for sleep disorders, including PLMS and restless legs syndrome. PSG performs tests such as electroencephalogram (EEG), electroencephalogram (EOG), electrocardiogram (ECG), and electromyography (EMG) by attaching various types of equipment to the patient’s body to measure vital signs. In addition, PSG can accurately and objectively diagnose all sleep-related disorders based on bio-signals recorded during sleep [7]. However, PSG is expensive, inconvenient, and depends on sleep experts. Most importantly, it is a time-consuming task as the sleep expert has to manually annotate the PLMS, and the results of the annotation may vary depending on the sleep expert’s experience and proficiency.

Electromyography (EMG) is an essential or standard physiological signal for the automatic detection of PLMS. Conventional studies have proposed rule-based models, machine learning algorithms, and deep learning models for automatic detection of the PLMS using EMG signal. Wetter et al. [8] invented the rule-based model of automatic detection of PLMS using a voltage threshold and time bridging of EMG signal. Ferri et al. [9] proposed a novel rule-based approach based on a double threshold to detect PLMS after rectification. Recently, Moore et al. [10] studied a machine learning algorithm applied to ECG and EMG signals for PLMS detection. They used adaptive signal processing for enhancing the ECG interference, EMG noise filtering, and adaptive thresholds. Cavelli et al. [11] proposed a deep learning model for PLMS scoring based on the convolutional neural network (CNN) and long short-term memory (LSTM) applied to EMG signals. However, there are no studies on deep learning for the automatic detection of PLMS using ECG signals only.

Several studies have proposed alternative methods for scoring PLMS based on various sensors such as accelerometer, bands, and film sensors [12,13,14,15]. King et al. [13] presented a novel approach to measuring PLMS using Actiwatch (Cambridge Neurotechnology Ltd., Cambridge, UK). They showed the promise of continuously measuring PLMS for monitoring and treatment. Prill and Fahrenberg [14] investigated how to evaluate PLMS using multiple accelerometers. However, these studies required additional devices or multiple sensors and their performance was insufficient. Finally, Wetter et al. [8] developed an automatic scoring method based on EMG signals during PSG. They achieved an accuracy of 92.5% in detecting PLMS episodes. Portable or home PSGs are essential for home sleep monitoring to diagnose sleep disorders [15]. The portable PSG requires only four channels for physiological signals such as ECG, respiration, SpO2, and photoplethysmography. Therefore, new algorithms based on single-lead ECG signals are needed for PLMS monitoring in laboratory and home settings to provide easy and accurate healthcare solutions.

In this study, our contribution or novelty is the easy and accurate model for the automatic detection of the PLMS events. For easy implementation, a single-lead ECG-based detection will be demonstrated based on a deep learning algorithm. To achieve reliable results, a deep learning algorithm was designed with optimal architecture and trained real clinical datasets. Finally, we propose a novel approach for automatic detection of PLMS patients using a deep learning model using single-lead ECG signals. The proposed model is called deepPLM and it consists of CNN and LSTM with optimized architecture and parameters for PLMS detection from ECG signal. Clinical data sets obtained from control and patient groups with PLMS were used for performance evaluation of the training and testing phases.

## 2. Materials and Methods

### 2.1. Study Population

**Figure 1:**
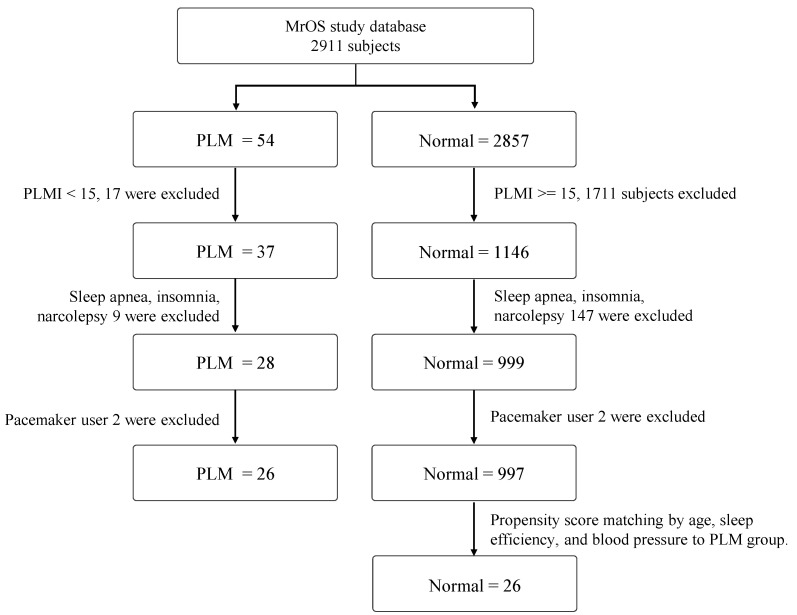
The exclusion criteria of the study population for the proposed deepPLM.

For this study, data from the Osteoporotic Fractures in Men (MrOS) Sleep Study, which was conducted as an auxiliary study for osteoporotic fractures, was utilized. The MrOS Sleep Study examined 5994 men aged 65 years or older between 2000 and 2002 [16]. Out of these participants, PSG was conducted on 2911 people. The following exclusion criteria was applied to all PSG subjects. The exclusion criteria were subjects with a periodic leg movement index (PLMI) of less than 15 in the patient group and a PLMI of 15 or more in the normal group were excluded. Then, the subjects were excluded who were diagnosed with sleep disorders including sleep apnea, insomnia, and narcolepsy. Finally, we excluded subjects who currently use the pacemakers (Figure 1).

Table 1 summarizes the clinical characteristics of the study subjects who passed the exclusion criteria.

### 2.2. ECG Dataset

The ECG signals were extracted from the PSG records of all subjects and stored at a sampling rate of 517 Hz. The analysis signal was an ECG signal with an average length of 2.8 h after each subject’s sleeping onset, and the total analysis length was 144.6 h. The entire ECG was segmented into units of 10 s and consisted of 5120 samples per segment. In the dataset, the ECG section of the normal group was indexed as 0, that of the patient group was indexed as 1, and the ratio of 0 to 1 was 1:1. For the training and evaluation of the proposed deepPLM model, the entire dataset was divided into a training group (66,560 segments from 32 subjects), an evaluation group (16,640 segments from 8 subjects), and a test group (20,800 segments from 12 subjects). The distribution of each dataset for normal and PLM events is represented in Table 2.

### 2.3. DeepPLM Model

**Figure 2:**
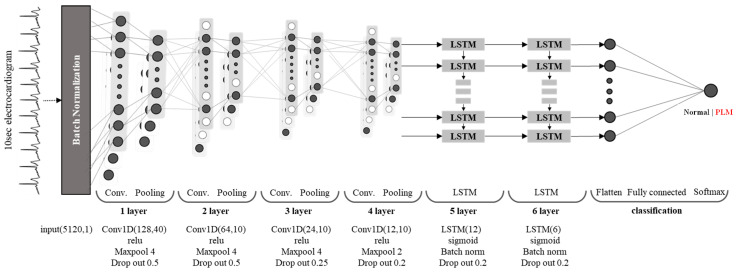
Architecture of the proposed deepPLM for the automatic detection of PLMSs.

In this study, the deepPLM model was constructed and optimized for the automatic detection of PLMS from a single-lead ECG since PLMSs result from several complex causes and the ECG signal contains information about movement, respiratory, and cardiac activities. We intended to develop and validate a deep learning model that can train and learn from the single-lead ECG to distinguish between PLMS and non-PLMS. Therefore, the deepPLM model is composed of a combined structure of the deep neural network that uses a CNN and LSTM, a recurrent neural network (RNN). First, a CNN was stacked in four layers and used to automatically extract a feature map by learning the pattern of the input signal [17]. Then, to control the periodic correlation or long-term dependence on the extracted feature maps, LSTM was connected in a two-layer structure. The final judgment was performed by configuring the complete connection layer and the soft-max function. Figure 2 illustrates the configured deepPLM model in detail.

The proposed deepPLM model has been implemented by one-dimensional (1D) convolutional layers and LSTM units to accurately detect the PLMSs from the ECG segments. In addition, the proposed deepPLM model was optimized using batch-normalization [18], dropout [19], and activation function [20] not only to avoid over and underfitting, but also to achieve robust detection performances.

All ECG input is applied to the batch-normalization as a pre-processor before training the configured deepPLM model. Batch-normalization is implemented by Equation (1).

(1) $$x_{b}=\alpha \cdot \left(\frac{x_{i}-\mu }{\sqrt{\sigma^{2}+\varepsilon }}\right)+\beta$$

where *ε*—random noise, *μ*—mean of mini-batches, *σ*—variance of mini-batches, *α*—scale parameter, and *β*—shift parameter. Both *α* and *β* are trainable and updated in an epoch-wise manner.

Since input ECG segments are selected from the physiological signal and shaped as 1D time series, a 1D convolutional layer is appropriate to extract features by high order data abstractions [21]. A 1D convolution layer was implemented by the 1D convolution operation that is faster than two or three-dimensional convolutions, and it can be simply expressed the following equation:

(2) $$x_{k}=b_{k}+\sum_{i=1}^{N}w_{k}\times y_{i}$$

where *x_k_* is the *k*-th feature map, *b_k_* is the bias of the *k*-th feature map, *w_k_* is the *k*-th convolutional kernel from all features of the *k*-th feature map, and *y_i_* represents the *i*-th feature map.

The pooling layer was used after each convolutional layer to reduce the dimensions of the intermediate feature maps. The max-pooling approach and appropriate parameters were applied in the pooling layers.

LSTM is an updated version of simple RNN with memory cells to make learning temporal associations easy over the long duration. [22]. LSTM consists of the three main memory cells which are called gates: an input gate, an output gate, and a forget gate [23,24]. LSTM is expressed as follows.

The input gate controls the flow of input activations into the memory cell.

*i_t_* = σ(*W^xi^x_t_* + *W^hi^h_t_*_−1_ + *b_i_*). (3)

The output gate controls the output flow of cell activations into the rest of the network.

*o_t_* = *σ*(*W^xo^x_t_* + *W^ho^h_t_*_−1_ + *b_o_*). (4)

The forget gate scales the internal state of the cell before adding it as input through the self-recurrent connection of the cell. Therefore, it adaptively forgets or resets the cell’s memory.

*f_t_* = *σ*(*W^xf^x_t_* + *W^hf^h*_*t*−1_ + *b_f_*), (5)

*g_t_* = *σ*(*W^xc^x_t_* + *W^hc^h*_*t*−1_ + *b_c_*), (6)

*c_t_* = *f_t_* × *c*_*t* – 1_ + *i_t_* × *g_t_*, (7)

*h_t_* = *o_t_* × *ϕ*(*c_t_*), (8)

where *i*, *f*, *o*, and *c* are respectively the input gate, forget gate, output gate, and cell activation vectors, all of which are the same size as vector *h*, defining the hidden value. Terms *σ* and *τ* represent nonlinear and hyperbolic tangent functions, respectively.

Lastly, some optimization techniques such as dropout and rectified-liner unit (ReLU) were applied to the configured deepPLM model. Dropout is a technique of randomly dropping out the nodes in a network to reduce overfitting by preventing complex adaptations on training data in the network [19]. ReLU is an activation function known as the robust training performance and consistent gradients, thereby facilitating gradient-based learning [25]. ReLU can be represented as

(9) f(x)=max(0,wx+b)

where *x* is the feature map, *w* is the weight, and *b* is the bias.

### 2.4. Implementation

In this study, data selection was performed using the statistical software R, and the pre-processing of PSG data was conducted using Python 3.7.4. The deepPLM model was designed based on the Keras [26] and TensorFlow [27] frameworks. To train and test the deepPLM model, we used hardware embedded with a graphics processing unit (GPU), the GeForce GTX 1080 Ti (11 GB, GDDR5X). The model performance was compared with that of a model built with a batch size of 16 and 500 epochs of iterative learning [28].

### 2.5. Evaluation Index

To evaluate the performance of the deepPLM model using a single-lead ECG signal, the following evaluation measures were used: precision, recall, and F1-score. To obtain the F1-score, two evaluation measures, precision and recall, were combined. These are defined as follows:

(10) $$precision=\frac{\mathrm{TP}}{\mathrm{TP}+\mathrm{FP}}$$

(11) $$recall=\frac{\mathrm{TP}}{\mathrm{TP}+\mathrm{FN}}$$

where TP, FP, and FN represent the true positive, false positive, and false negative, respectively. They are determined for each sleep stage event.

The F1-score, better known as the unbalanced dataset, is computed based on the sample proportion of precision and recall as follows:

(12) $$F1=2\times \frac{precision*recall}{precision+recall}$$

## 3. Results

### 3.1. Performance of the Single-Lead ECG-Based Detection

This study demonstrated a single-lead ECG-based approach for the automatic detection of PLMS. The results showed very high performances for the automatic detection of PLMSs based on the single-lead ECG signal (Table 3). For the evaluation measures, we used many indexes including the precision, recall, accuracy, and F1-score. Among them, the F1-score of the deepPLM model was found to be 96% for the training set and 92% for the validation and test sets. In addition, the detection accuracy was high, at 88% for the training group, 92% for the evaluation group, and 91.5% for the test group.

**Figure 3:**
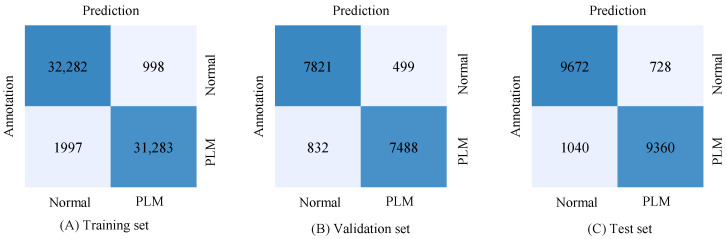
Confusion matrix of the proposed deepPLM model for the automatic detection of PLMS: (**A**) training set, (**B**) validation set, and (**C**) test set.

Figure 3 shows the evaluation results of the proposed deepPLM model in a confusion matrix for each dataset.

### 3.2. Performance of the DeepPLM Model Optimization

**Figure 4:**
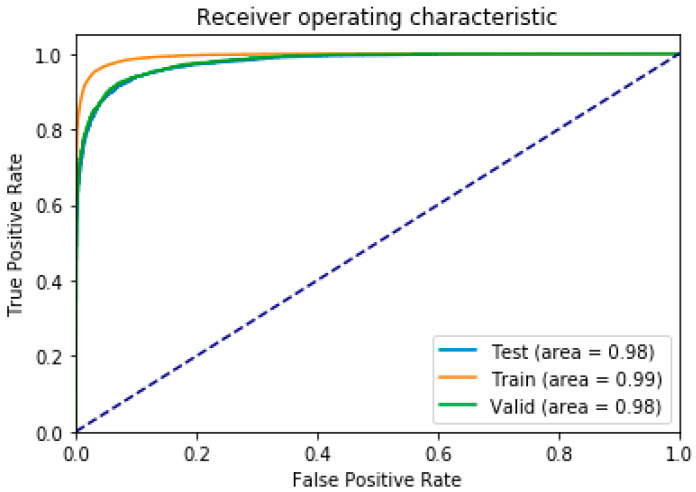
ROC and AUC of the proposed deepPLM model for the automatic detection of PLMS.

The proposed deepPLM model has a simple design and well optimizer. ROC and AUC were used to these characteristics of the deepPLM for automatic detection of PLMS. Figure 4 shows the evaluation results via the ROC curve, which indicates that the AUC value is very high at 99% for the training group, 98% for the evaluation group, and 98% for the test group.

## 4. Discussion

This study proposes a novel approach based on deep learning for the automatic detection of PLMS in patients using a single-lead ECG signal. Deep learning was constructed, optimized, and named as the deepPLM model, and it was evaluated using clinical data sets measured from the control group and patients with PLMS from the MrOS database. The training group achieved high performance with an accuracy of 88%, the evaluation group achieved an accuracy of 92%, and the accuracy test group achieved an accuracy of 91.5%. The results showed a possibility of the automatic detection of PLMS from single-lead ECG using a deep learning model.

PLMS is considered prevalent among people over the age of 65. However, predictive methods for PLMS patients are not yet available [29]. Because of motion artifacts in ECG signals, the proposed approach can accurately distinguish between normal and abnormal ECGs for deep learning-based automatic detection of PLMS. ECG signals are an essential and important source of clinical information for sleep laboratory nighttime PSG as well as home sleep monitoring using portable PSG. The proposed approach for automatic detection of PLMS provides a simple deepPLM architecture that can be easily implemented with real world data. The proposed approach shows advantages such as robust performance, simultaneous usability in laboratory and home sleep monitoring, and easy implementation. First, the results have shown the possibility of the automatic detection of the PLMS from only short-term ECG segments. Since the PLMS is automatically detected, it can be used in prescreening for the associated diseases, including the cardiovascular diseases [30], cerebrovascular risks [31], and Parkinson disease [32]. Second, we studied an alternative model for automatic detection of PLMS based on deep learning from the single-lead ECG signal. The proposed deepPLM model can be used as an easy screening tool based on PSG or portable ECG that does not use the phenotypes and clinical parameters. Finally, we achieved a higher detection performance than similar conventional studies listed in Table 4.

Table 4 summarizes comparisons with previous related studies on scoring or detecting PLMS. The proposed deepPLM model achieved the best performance for the automatic detection of PLMS, which outperforms the related previous studies. Conventional studies proposed a rule-based method such as voltage thresholding and time bridging of the EMG signal [8], and double thresholding techniques after rectification [9]. However, these presented a lower performance for the PLMS detection. Another study proposed adaptive signal processing methods for PLMS detection from EMG signals. The authors used adaptive processing to the ECG interference, noise cancelation, and adaptive thresholds for PLMS detection from EMG signals [10]. Finally, Cavelli et al. [11] proposed a deep learning model for PLMS scoring using CNN and LSTM similar to the proposed model. All these studies used EMG signal for PLMS detection, and they cover the rule-based model, machine learning, and deep learning algorithms. However, the results were shown to be lower than the proposed deepPLM model which used single-lead ECG signals.

Nevertheless, this study has limitations in that it requires a small amount of data and high computational power. For this study, we used ECG signals from 52 subjects. Future studies should use a larger study population to overcome these limitations. To compute high-dimensional data abstractions, deepPLM models require relatively higher computational power than traditional machine learning methods. Therefore, in this study, a CNN–LSTM model was constructed and optimized with a simple and small structure.

In summary, we propose a novel method for automatic PLMS prediction in patients using a deep learning model with ECG signals. The deepPLM model was constructed and evaluated using datasets from control and PLMS patients. A high satisfactory performance was obtained on the training and test sets. We also propose an alternative method to predict a patient’s PLMS. Our results demonstrate the feasibility of using deep learning models with ECG signals for the automatic detection of PLMS. The results also demonstrate that the single-lead ECG signal can be used as a discriminant and alternative signal for patients with PLMS. The proposed approach is sufficient and can be a useful predictive tool for detecting sleep-related movements, including PLMS. Further studies will require a more diverse patient group and larger data sets to confirm and support these findings.

## Figures and Tables

**Table 1:** Demographics of the study population.

Characteristics	Normal	PLM
Subjects (*N*)	26	26
Age (years)	76.12 ± 5.51	76.08 ± 5.11
Periodic leg movement index (per hour)	2.46 ± 4.16	57.88 ± 30.27
Body mass index (kg/m^2^)	27.92 ± 3.12	29.15 ± 3.89
Sleep efficiency (%)	74.35 ± 10.93	73.00 ± 11.34
Smoking status, *n* (%)		
Never Past	12 (47.15%) 14 (53.85%)	12 (56.0%) 14 (40.0%)
Blood pressure		
Systolic Diastolic	127.57 ± 12.82 66.85 ± 5.66	127.35 ± 19.08 68.81 ± 7.35

**Table 2:** ECG dataset information.

Datasets	Normal	PLM	Total
Training set	33,280	33,280	66,560
Validation set	8320	8320	16,640
Test set	10,400	10,400	20,800
Total	52,000	52,000	104,000

**Table 3:** The performance of the deepPLM model for the automatic detection of PLMS patients.

Datasets	Segment	Precision	Recall	F1-Score	Accuracy
Training set	Normal	0.94	0.97	0.96	0.89
	PLM	0.97	0.94	0.96	
Validation set	Normal	0.90	0.94	0.92	0.92
	PLM	0.94	0.90	0.92	
Test set	Normal	0.90	0.93	0.92	0.92
	PLM	0.93	0.90	0.92	

**Table 4:** Comparison with previous related studies.

Authors (Year of Publication)	No. of Subjects	Signal	Method	Results (F1-Score)
Wetter et al. (2004) [8]	24	EMG	EMG-based analytical method	0.63
Ferri et al. (2005) [9]	30	EMG	Computer-assisted detection method	0.72
Moore et al. (2014) [10]	1833	EMG, ECG	Ten-step PLM detection method	0.79
Carvelli et al. (2020) [11]	800	EMG	CNN–LSTM model	0.85
This work	52	ECG	CNN–LSTM model	0.92

## Data Availability

https://sleepdata.org/datasets/mros. All data is approved by the National Sleep Research Resource (NSRR) for the specific purpose of this study.
